# Rare case of nephrocalcinosis in a 14-year-old girl: Answers

**DOI:** 10.1007/s00467-016-3440-3

**Published:** 2016-07-06

**Authors:** Omar Bjanid, Piotr Adamczyk, Małgorzata Stojewska, Dagmara Roszkowska-Bjanid, Magdalena Paszyna-Grześkowiak, Agnieszka Jędzura, Joanna Oświęcimska, Katarzyna Ziora, Aurelia Morawiec-Knysak, Maria Szczepańska

**Affiliations:** 1 Dialysis Division for Children, Department of Pediatric Nephrology, Public Clinical Hospital No. 1 in Zabrze, ul. 3 Maja 13/15, 41-800 Zabrze, Poland; 2 Chair and Clinical Department of Pediatrics, School of Medicine with the Division of Dentistry in Zabrze, Medical University of Silesia in Katowice, ul. 3 Maja 13/15, 41-800 Zabrze, Poland

**Keywords:** Hypoparathyroidism, Hypercalciuria, Nephrocalcinosis, Autoimmune polyendocrine syndrome type 1, Calcium supplementation

## Question 1. What is the final diagnosis?

Hypoparathyroidism in childhood is predominantly due to congenital parathyroid agenesis or hypoplasia, associated with numerous conditions, the most common one being the Di George syndrome (approximately 70 % of children with isolated hypoparathyroidism) [1]. Rare conditions may include specific renal disturbances, such as steroid-resistant nephrotic syndrome, kidney dysplasia, hypoplasia or aplasia in Barakat syndrome [2]. From a nephrologist perspective, it is also worth noting that hypomagnesemia (e.g. during chronic cyclosporine or diuretic treatment) inhibits parathyroid hormone secretion and can mimic hypoparathyroidism.

In the presented case, however, the coexistence of hypoparathyroidism, oral candidiasis, dental enamel hypoplasia and subclinical Hashimoto's disease is strongly suggestive of autoimmune polyglandular syndrome (APS) type 1. One of the clinical implications of this diagnosis is the high probability of future occurrence of adrenal insufficiency, emphasizing the importance of maintaining a high level of suspicion with the onset of symptoms such as weakness, fainting, hypotonia or hyperkalemia. Addison’s disease would in fact represent quite a challenge in terms of the future management of this patient.

## Question 2. What is the cause of nephrocalcinosis?

**Fig. 1:**
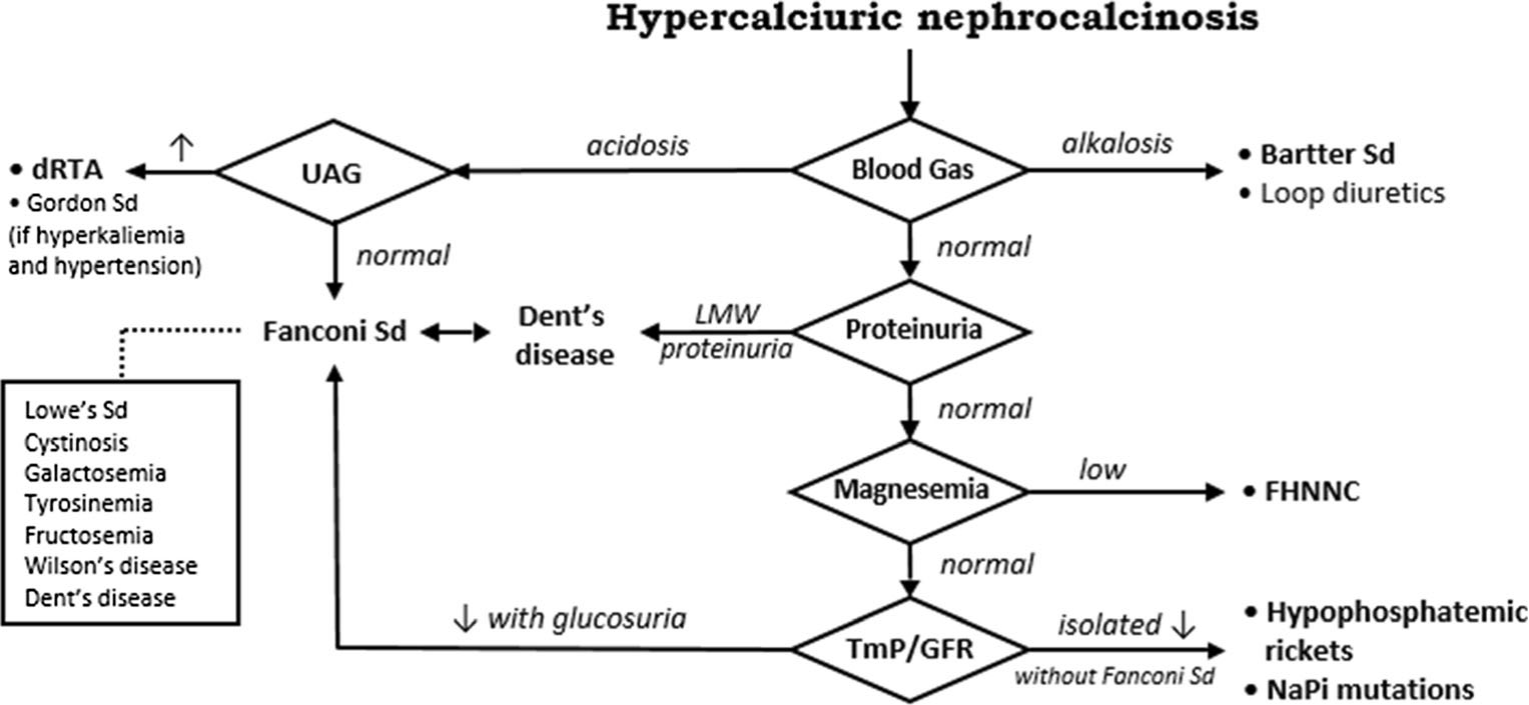
Tubular disorders with hypercalciuric nephrocalcinosis. *dRTA *Distal renal tubular acidosis, *UAG* urine anion gap, *Sd* syndrome, *LMW* low molecular weight, *FHHNC* familial hypomagnesemia with hypercalciuria and nephrocalcinosis,*TmP/GFR* renal tubular maximum reabsorption rate of phosphate to glomerular filtration rate, *NaPi* sodium/phosphate cotransporter

Nephrocalcinosis is defined as radiologically detectable deposits of calcium in the renal cortex, medulla or both. The most severe form is a generalized calcification of kidneys seen in classical imaging studies, including renal ultrasonography [3]. A metabolic risk factor is identifiable in about 75 % of children with nephrocalcinosis. In addition to hyperoxaluria and hypercystynuria, both of which are relatively rare but none-the-less important due to their potential morbidity, hypercalciuria stands out by its frequency. Three basic pathophysiological alterations may play a role in hypercalciuria: increased intestinal absorption, increased bone release and decreased renal tubular reabsorption of calcium. Almost every hypercalcemic state, such as hyperparathyroidism, Williams–Beuren syndrome or vitamin D intoxication, can promote nephrocalcinosis [4]. The same applies to bone disorders, including the effect of acidosis on the release of bone buffers and calcium. Non-hypercalcemic tubular disorders with hypercalciuric nephrocalcinosis are recapitulated in Fig 1. They were ruled out in our patient.

The problem of an excessive dietetic load of calcium commonly arises in chronic supplementation which is the usual therapy for disorders like hypophosphatemic rickets or hypoparathyroidism. The therapeutic window is often narrow because of the renal calcium leak, and it is difficult to reach the optimal bone–kidney trade-off. The etiology of hypoparathyroidism may also predispose to the development of nephrocalcinosis, as hypercalciuria is typically more pronounced in patients with activating mutations of the calcium sensing receptor [5]. Winner et al. described the presence of nephrocalcinosis in the course of hypoparathyroidism of various etiologies in >40 % of patients after 20 (or more) years of the disease [6]. In our patient, it is difficult to rule out an additional factor, such as idiopathic hypercalciuria, as well as autoimmune or infectious (e.g. fungal) gastrointestinal disorders that are common in APS type 1 and can cause important fluctuations in calcium absorption, narrowing even more the therapeutic window. However, it is likely that the main cause of nephrocalcinosis is iatrogenic and linked to the relatively high doses of calcium and vitamin D supplementation, and probably also the incident of acute hypercalcemia treated with loop diuretics. In fact, the presented case is a typical example of so-called refractory hypoparathyroidism, which refers to the difficulty to achieve normocalcemia, the need for high-dose calcium carbonate treatment and the frequent fluctuations with episodes of transient hypercalcemia [7]. Such problematic management is typically observed in such disorders as APS type 1, hypoparathyroidism associated with malabsorption (especially celiac disease), and activating mutations of the calcium sensing receptor (CaSR) [5]. Those patients are understandably at a higher risk of chronic kidney damage.

## Question 3. What further treatment options are available?

Substitution therapy in the treatment of APS is similar to that in single endocrine organ failure. Calcium supplementation in hypoparathyroidism is best achieved orally in three to four divided daily doses, (20 mg/kg/day up to 1 g/day) combined with 1,25-dihydroxyvitamin D or 1-α-hydroxyvitamin D (average 0.03 μg/kg/day). Magnesium levels should be closely watched and supplemented usually at a dose of 50–200 mg daily if needed [8]. It is also important to restrict the phosphorus in the diet. Regular check-ups of plasma and urine calcium is recommended to adapt the dosage adequately to keep calcemia at the lower normal range and calciuria at <4 mg/kg/24 h. If the therapeutic goals are not achieved, the use of thiazide diuretics can be helpful. Thiazides reduce the calcium renal leak and calciuria, while simultaneously increasing calcemia, thus widening the therapeutic window and allowing calcium carbonate and vitamin D dose reduction. Moderate hyperphosphatemia is generally not a problem provided blood calcium levels are maintained at the lower normal range and the calcium × phosphate product falls within safe limits.

A treatment with 12.5 mg/24 h of hydrochlorothiazide in the presented patient significantly decreased the urine Ca/creatinine ratio to 0.19 mg/mg. Restriction of dietetic phosphate and salt and supplementation with magnesium citrate were continued. An attempt to liberalize the diet was made, but it resulted in worsening of the hypercalciuria and an increase in phosphatemia. The dose of hydrochlorothiazide had to be increased to 25 mg/24 h (in divided doses) to maintain a sustained hypocalciuric effect. As a result of hypokalemia (3.68 mmol/l) secondary to hydrochlorothiazide treatment, potassium chloride supplements were added to the therapeutic regimen. With this additional treatment all therapeutic goals and stable calcium parameters were achieved with ultimately 3 g of calcium carbonate and 1 μg daily of alfacalcidiol.

Despite this encouraging outcome at the present time, a potential complication is to be expected. APS type 1 is characterized by the successive onset of three cardinal symptoms: the first to appear is usually candidiasis, the second is hypoparathyroidism and the third is Addison's disease with a peak incidence in teenagers aged between 10–15 years.

Although adrenal insufficiency is treatable with substitution of hydrocortisone and analogues of endogenous mineralocorticoids, the condition may lead to recurring renal salt losing with serious general symptoms, such as hypotonia and loss of consciousness. Therapy with thiazide diuretics can obviously aggravate this problem through impairment of tubular salt reabsorption in the distal part of the nephron. Adrenal insufficiency is therefore considered to be a relative contraindication to thiazide treatment; however, the withdrawal of thiazides will probably result in an exacerbation of the hypercalciuria and progression of nephrocalcinosis, which can undoubtedly be perceived as a risk factor for progressive chronic kidney damage. The expected fully symptomatic presentation of APS type 1 (including renal insufficiency) will therefore make the treatment of all combined disturbances more and more challenging. Only strict monitoring based on repeated laboratory tests will allow correction of the primary symptoms caused by the disease per se, without any increase in the risk of iatrogenic disturbances, which potentially may be even more harmful than uncorrected disorders.

## Commentary

This clinical quiz highlights the importance of careful evaluation of all multiorgan symptoms occurring in the described patient to prevent further complications.

In 1980, Neufeld and Blizzard proposed the first classification of autoimmune polyendocrine syndromes on the basis of clinical criteria and defined four types of APS [9]. Of these four types, polyglandular autoimmune syndrome type 1, also known as APS1, autoimmune polyendocrinopathy–candiasis–ectodermal dystrophy or Whitaker syndrome, is a rare genetic disease which is autosomal recessive, inherited in a monogenic pattern and caused by a mutation in the gene of autoimmune regulation (*AIRE*) localized on the long arm of chromosome 21 (21q22.3) [OMIM 240300] [10, 11]. It was described for the first time in 1946 by Leonard [12]. The prevalence of this syndrome in the Polish population was set at 1:129 000 people. APS1 occurs in both sexes. The suggested role for AIRE is to induce expression of peripheral antigens in the thymus, thereby mediating negative selection of autoreactive T cells and cause toleration [13]. The main components of APS1 are hypoparathyroidism, adrenal insufficiency and candidiasis of mucous membranes (oral, anal area, external genitals) and/or of the skin and nails. A necessary condition for the diagnosis of APS1 is the simultaneous occurrence of two of the three above-mentioned diseases. APS1 is accompanied by other diseases with an autoimmune origin [14–16]. The first symptom is usually candidiasis of the oral mucosa, which occurs before the age of 5 years. Autoimmunity in APS1 is also the responsible factor for the *CYSTATIN salivary antigen1* deficiency, which causes impaired protection against *Candida albicans* infections [17]. The first endocrinopathy is an autoimmune hypoparathyroidism, which occurs before the age of 10 years, and the second is adrenal insufficiency, which usually develops before the age of 15 years [9, 18].

Additional disorders include other endocrinopathies (autoimmune hypothyroidism, type 1 diabetes, hypergonadotropic hypogonadism, hormonal disorders of pituitary origin), ectodermal dystrophy, including tooth enamel hypoplasia, abnormal development of nails, gastrointestinal diseases (chronic atrophic gastritis, malabsorption symptoms, cholelithiasis), autoimmune hepatitis, alopecia areata, vitiligo, sclerositis and conjunctivitis, humoral and cellular immunity deficiency, asplenia and anemia associated with B12 deficiency [8, 19–21].

In patients with APS1 it is necessary to perform periodical screening for 21-hydroxylase antibodies, anti-islet cell antibodies, antiparietal cell antibodies, transglutaminase antibodies and vitamin B12 deficiency. Autoantibodies to the T helper 17 cells and anti-cytokines interleukin (IL)-17A, IL-17 F and IL-22 appear in APS1 patients and may explain their increased susceptibility to candidal infections [22, 23].

The occurrence of several additional symptoms may be promoted by the overlap between the initial disorders and some effects of the therapeutic interventions. Among these secondary symptoms, basal ganglia calcification and the already discussed nephrocalcinosis should be mentioned. Chronic hyperphosphatemia as a primary disturbance in combination with excessive calcium and supplementation of active vitamin D analogues contributes to the ectopic calcifications in the central nervous system, most commonly in the globus pallidus [24]. These calcifications are believed to be primarily the direct consequence of chronically elevated phosphate levels and high calcium × phosphate product. Nonetheless, additional factors explaining, among others the brain tropism, are taken into consideration. For example, the high serum phosphate levels may activate the inorganic phosphate transporter (PiT1; SLC20A1), which results in the expression of osteogenic molecules in the caudate nucleus and gray matter of the brain [25, 26]. It is estimated that cerebral calcifications may be present in >50 % of patients with hypoparathyroidism. These calcifications can remain asymptomatic or lead to progressive clinical symptoms, including cognitive impairment, extrapyramidal symptoms and psychosis [27].

Consequently, because basal ganglia concretions may result from “overtreatment” of hypoparathyroidism, it is important to set the therapeutic goal so as to avoid symptomatic hypocalcemia, without constant striving for full biochemical normalization of calcemia.

Urinary calcium excretion in APS1 may vary widely between individuals and is highly dependent on treatment, medication compliance and diet. As in nephrocalcinosis of other origins, thiazide diuretics have also been successfully applied in APS1. Thiazide diuretics have been shown to reduce urinary calcium excretion and thus reduce the formation of calcium deposits. Thiazides diminish renal calcium excretion by increasing calcium reabsorption in the distal tubules and by stimulating proximal tubular reabsorption by way of volume control. Thiazides also reduce gastrointestinal absorption of calcium and phosphorus. The final result of these is to promote a positive calcium balance in the body [28]. Hypokalemia is an unwanted side effect that can lead to hypocitraturia and an unplanned increase of the urinary saturation (e.g. for calcium oxalate). In addition, thiazide medication may also lead to lowering of the blood pressure [29, 30]. Auron and Alon reported the resolution of medullary nephrocalcinosis in three children with metabolic bone disorders following treatment with thiazides [29]. Tubulointerstitial nephritis has also been mentioned as a symptom of APS1. Kluger et al. propose regular kidney monitoring for APS1 patients as they may present with kidney injury due to the presence of circulating antibodies against the distal part of the nephron [31].
